# Initial experiences using plasma rich in growth factors to treat keratoneuralgia

**DOI:** 10.3389/fmed.2022.946828

**Published:** 2022-08-24

**Authors:** Margaret Wang, Sowmya Yennam, Stephen Pflugfelder

**Affiliations:** Department of Ophthalmology, Baylor College of Medicine, Houston, TX, United States

**Keywords:** cornea nerve, pain, LASIK (laser *in situ* keratomileusis), plasma, dry eye

## Abstract

Keratoneuralgia, a clinical diagnosis of sensitized corneal pain without visible ocular surface damage, generally has minimal response to conventional therapies. Causes include refractive surgery and chronic dry eye. We evaluated the efficacy of Plasma Rich in Growth Factors (PRGF), a novel treatment prepared using a commercially available kit, in patients with keratoneuralgia. A retrospective chart review identified patients who had the clinical diagnosis of keratoneuralgia and were treated with PRGF for at least 3 months from October 2015 to April 2020 at a single academic institution. Both objective eye exam findings and concurrent treatments were obtained at baseline, 3 months, and final visit (if available). A questionnaire was administered to identified patients, including symptoms scores measured with a visual analog scale. The results of this survey and other objective findings were compared before and after PRGF treatment. 16 out of 32 patients (50%) with a mean follow-up period of 33 ± 26 months answered the questionnaire. Refractive surgeries were the cause of keratoneuralgia in 14 patients (87.5%), with LASIK the most common procedure (11 patients, 69%). There were no adverse events recorded or reported. Symptom scored by VAS in a modified Symptoms Assessment in Dry Eye questionnaire significantly decreased after PRGF use (85 ± 16 to 45 ± 33, *p* = 0.0002). Ten patients (63%) reported PRGF is superior to other therapy and would recommend to others. There were no significant trends in visual acuity, objective exam findings, or concurrent treatments after PRGF treatment. PRGF is safe and can potentially alleviate symptoms in patients with keratoneuralgia, a rare but devastating complication after refractive surgery. Prospective trial is indicated to explore PRGF as a potentially useful treatment for keratoneuralgia.

## Introduction

Keratoneuralgia is a clinical diagnosis of sensitized corneal neuropathic pain presenting without visible ocular surface damage. Occurring most commonly in younger patients with a history of refractive surgery, chronic dry eye disease, or in setting of infectious or inflammatory processes, it can present with chronic pain, photoallodynia, burning, irritation, dryness, and grittiness (1, 2). Keratoneuralgia is thought to result from the sensitization of peripheral axons after injury or disease of the corneal nerves (1). Over time, this peripheral sensitization can become centralized, resulting in severe allodynia or hyperalgesia unresponsive to topical therapies (3). The somatosensory etiology results in symptoms out of proportion to observed signs of disease or damage seen on the ocular surface, giving rise to the name “pain without stain” (4).

Current management strategies for keratoneuralgia differ by source of neuropathic pain. Proposed first-line agents include tricyclic antidepressants (TCAs) and anticonvulsant carbamazepine (CBZ) (1). In cases of peripheral sensitization, a combination of local and systemic therapies can be applied in an endeavor to suppress inflammation, protect the ocular surface, suppress nerve sensitization and promote neuro-regeneration (1, 5). Topical corticosteroids may improve symptoms, but they can increase intraocular pressure and carry risk of cataract formation (6). Treatment with therapies such as preservative-free artificial tears, punctal plugs, or contact lenses, may improve ocular surface signs, but often provide minimal to no improvement in pain symptoms after prolonged use (1, 5, 7). Autologous serum tears (AST), as a neuro-regenerative therapy, has been shown to increase nerve density and regeneration in a cohort of corneal neuropathy-induced photoallodynia and a separate group of patients with neuropathic eye pain (8, 9). It has also been postulated that nerve growth factor can be a potential treatment for neuropathic pain (1).

Plasma rich in growth factors (PRGF) is a novel leukocyte-free autologous plasma formulation containing biologically active constituents, including platelet derived factors such as epidermal growth factor, transforming growth factor beta, and platelet-derived growth factor, found to suppress inflammation and fibrosis in various organ systems (eye, skin, joint, and dental) (10). Unlike other autologous plasma products, it has a standardized production protocol approved by the FDA and ensures a leukocyte-free formulation (11). Preclinical studies have shown its ability to decrease inflammation and promote ocular surface healing (12). Clinical efficacy has been demonstrated for patients with a number of ocular surface diseases including dry eye from a variety of causes and neurotrophic keratitis (13, 14). The efficacy of PRGF on keratoneuralgia has not been studied. The objective of our study is to review the safety and efficacy of topical PRGF in patients with keratoneuralgia.

## Materials and methods

This study was approved by the Baylor College of Medicine Institutional Review Board (IRB; Protocol number H-44364), and all research adhered to the tenets of the Declaration of Helsinki. A retrospective chart review of all patients who received PRGF eye drop treatment at the Alkek Eye Center from 2016 to 2021 was performed. Patients were included if they were clinically diagnosed with keratoneuralgia, corneal nerve pain, or otherwise had severe symptoms of dry eye without traditional dry eye signs (i.e., TBUT < 4, cornea fluorescein staining). Patients were excluded if they used AST in the 3 months prior to starting PRGF. Those meeting the inclusion criteria were contacted via mail to ask permission to participate in a telephone satisfaction questionnaire about their PRGF use. Informed consent was obtained over the phone. The questionnaire (Supplementary Table 1) incorporates a recalled Symptom Assessment in Dry Eye (SANDE) questionnaire in questions 2 (severity) and 3 (frequency), based on recall of pre and post treatment symptoms via a visual analog scale from 0 to 100. SANDE has shown reliability and repeatability in assessing dry eye symptom changes in multiple studies, and it is similar to the 0–10 numeric rating scale commonly used in pain assessment. Telephone and in-person surveys were conducted over a 6-month period starting in October 2020. Only those who responded to the phone questionnaire were included in the study.

Change in pain symptoms as measured by SANDE score before and after using PRGF for at least 3 months were collected and served as the primary outcome measure. Additional data were collected before and after use of PRGF, including other subjective questions per patient recall (Supplementary Table 1), best corrected visual acuity (BCVA), results of conventional dry eye tests, and the number of other treatments used. Initial SANDE scores were also recorded in some patients’ initial clinical visits. When possible, these chart review initial SANDE scores were collected to verify the internal validity of the recalled SANDE scores in the questionnaire.

A commercial kit was used to prepare PRGF eye drops (Endoret-PRGF kit^®^, BTI Biotechnology Institute, Vitoria, Spain) by a previously published method (15). Using a process including centrifugation and degranulation of platelets, sterile enriched plasma was dispensed into 32 dropper bottles to be used topically (up to 4 times per day) for up to 6 months.

Comparative statistical analyses of pre- and post-PRGF questionnaires, corrected visual acuity, number of concurrent treatments were performed. Statistical comparison of VA was performed after conversion of Snellen measurements to logMAR values. Statistical analysis, including statistical summaries and two-sample paired T tests assuming equal variance were performed using Microsoft Excel 2010 (Microsoft Corporation, Redmond, WA, United States). Statistical significance was set at a *p* value of <0.05.

## Results

**FIGURE 1:**
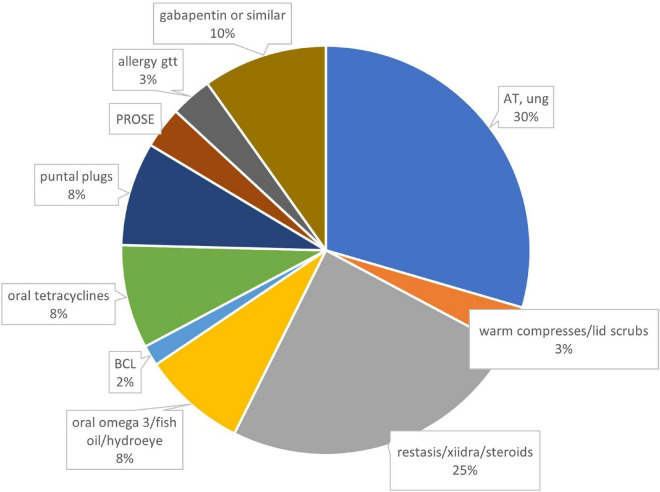
Therapies prior to using PRGF, by percentages. *N* = 78 treatments from 16 patients. AT, artificial tears; BCL, bandage contact lens; PROSE, prosthetic replacement of ocular surface ecosystem; gtt, drops; ung, ointment.

Sixteen out of 32 patients answered questionnaires (50% response rate), with average of 33 ± 26, median 22, range 4–72 weeks of follow-up. Half of the patients were female (Table 1). Systemic problems were noted in 4 patients which included: type 1 diabetes (1), migraine headache (1), Hashimoto’s thyroiditis and bladder spasm (1) and depression and sleep apnea (1). Fourteen of 16 patients attributed refractive surgery to their pain symptoms, with LASIK the most frequently performed procedure (Table 2). Initial symptoms prior to PRGF were severe, as measured by both the chart review initial SANDE scores out of 100 (*N* = 11, 89 ± 11, range 70–100) and recalled initial SANDE scores from the questionnaire (*N* = 16, 85 ± 16, range 46–100). There were no differences between the two initial scores for 11 patients who had both, suggesting consistency in patients’ recall (*p* = 0.378). Fifteen out of 16 patients had available clinical assessments of dry eye before starting PRGF, which did not indicate a typical diagnosis of dry eye (Table 3). Prior to PRGF, these 16 patients have tried an average of 5 ± 2 different therapies (range 2–9) over their treatment history before initiating PRGF to treat their recalcitrant pain. Artificial lubricants and topical anti-inflammatory agents were the most commonly used treatments before PRGF (Figure 1). No patients were recorded to have sustained pain after proparacaine administration, suggesting evidence of peripheral sensitization.

**TABLE 1:** Demographic information for the patients included in the study (*N* = 16).

Gender (female/males)	8/8
Average age, years ± SD (range)	52 ± 16 (27–70)
Females	56 ± 13 (29–69)
Males	48 ± 19 (27–70)
Ethnicity, *n* (%)	
Caucasian	12 (75)
Hispanic	1 (6)
Asian	2 (12)
Other	1 (6)

**TABLE 2:** History of refractive surgery and other ocular diseases.

Cause	*n* (%)
LASIK	11 (69)
PRK	1 (6)
RK	2 (12)
Other^a^	2 (13)

^a^Other includes a patient with history of SLK with no current staining and another patient with history of vitrectomy.

**TABLE 3:** Dry eye diagnostic test measurements before starting PRGF.

	Average ± SD (range)
TBUT (seconds)	5.2 ± 0.24 (3–9)
Cornea Fluorescein Staining (0–15)	1.1 ± 0.04 (0–3)
Schirmer 1 test (mm)	16 ± 1.1 (4–35)
Osmolarity (mOsm/L)	299 ± 0.41 (276–316)
OCT tear meniscus height (micrometers)	356 ± 32 (148–1,100)

N = 15.

OCT, optical coherent tomography.

**FIGURE 2:**
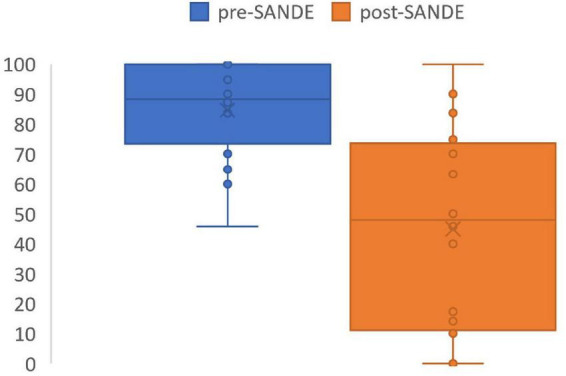
SANDE score before and after PRGF use. Pre-treatment SANDE mean 85 ± 16. Post-treatment SANDE mean 45 ± 33. *N* = 16. *P* = 0.0002.

SANDE scores significantly improved after PRGF use to 45 ± 33 out of 100 using the questionnaire (*p* = 0.0002, range 0–100, Figure 2). No side effects or complications were reported in the questionnaire, and 10 patients (63%) reported PRGF is better than any other treatment they have tried in the past (Table 4). Five out of the 16 patients had used AST in the past, all without improvement in symptoms. Three out of these five thought PRGF was better than any other drops, including serum drops. One patient preferred AST due to more ergonomic packaging and one patient does not recall a difference between the two autologous blood treatment modalities.

**TABLE 4:** Questionnaire results.

Question	Yes responses (%)
Still using PRGF	8 (50)
Reasons for no longer using	8 (50)
Does not work	4
Symptoms resolved	3
Did not answer	1
PRGF is better than other treatments?	10 (63)
Experienced side effects	0 (0)
Recommend to others?	11 (69)
Cost too high for value?	12 (75)
Cost preventing use of PRGF?	4 (25)

N = 16.

PRGF was well-tolerated by all patients and no side effects or adverse events to the treatments were reported.

**FIGURE 3:**
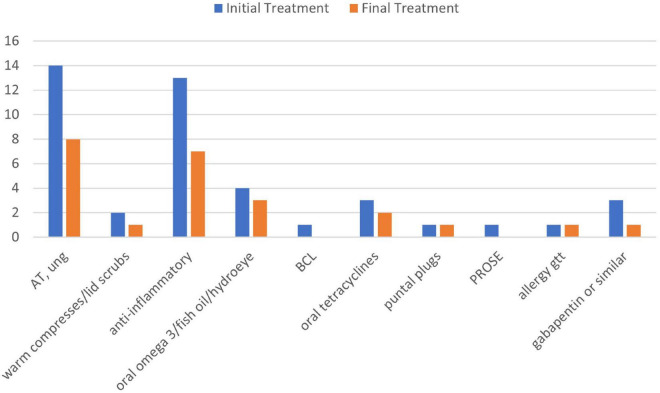
Treatments used before and after PRGF use. AT, artificial tears; anti-inflammatory, cyclosporine A 0.05%, lifitegrast 5%, and cortiosteroids; BCL, bandage contact lens; PROSE, prosthetic replacement of ocular surface ecosystem; gtt, drops; ung, ointment.

Ten out of 16 patients had objective measurements at least three months after their PRGF treatments, which were compared with their findings from before PRGF use. LogMar visual acuity before (0.021 ± 0.06, range -0.12 to 0.18) and after PRGF (0.013 ± 0.07, -0.12 to 0.18) were not significantly different (*P* = 0.75). There were no significant differences in medication burden before (3.8 ± 2, range 1–8) and after (3.4 ± 2, range 1–8 including PRGF) PRGF (*p* = 0.14). However, both lubricating and anti-inflammatory drops were stopped in almost half of the patients after PRGF use (Figure 3). There were not enough measurements of dry eye tests after PRGF use to establish a useful correlation.

Two of the 16 patients had confocal exams prior to starting PRGF that showed abnormal subepithelial plexi and presence of neuromas. This procedure was not repeated after therapy.

## Discussion

Treatment of keratoneuralgia is a challenge because patients with this condition complain of dry eye symptoms and pain, but typically experience minimal or no relief from conventional dry eye treatments (16). Rare but debilitating, keratoneuralgia has numerous causes, including trauma (corneal epithelial defect, chemical exposure (e.g., preservatives in topical medications, chemical burns, systemic chemotherapy), ultraviolet light and radiation exposure, herpes virus infection (herpes zoster and herpes simplex), eye surgery (refractive, cataract, glaucoma, and retinal surgery), systemic disease (autoimmune/inflammatory conditions, diabetes, fibromyalgia) and neurological disease (e.g., trigeminal neuralgia, migraine) (1–4). LASIK surgery is the most common because there is direct injury to the cornea nerve plexus during flap creation or laser ablation of the stroma (17).

Our study examined the subjective experiences of keratoneuralgia patients with PRGF therapy using a standardized questionnaire. This format of evaluating treatment efficacy especially suits these patients as objective ocular surface testing does not reflect the extent of keratoneuralgia suffered by patients. We were able to identify a small cohort of patients with clinically diagnosed keratoneuralgia in our 5 years of chart review who answered the questionnaire. These patients, equal in gender distribution, mostly attributed keratorefractive surgery to their pain, especially prior LASIK. They have the classic “pain without stain” as confirmed by scarce exam findings but high SANDE scores which were refractory to many conventional treatments. The SANDE questionnaires were collected at initial visits to discern the level of eye irritation/pain since most keratoneuralgia patients were referred with a diagnosis of dry eye. It is based on a visual analog scale very similar to the 10-point analog scale used by pain specialists (18–21).

The retrospective questionnaire showed statistically significant and dramatic findings. Symptom scores, as measured by the short but reproducible SANDE questionnaire, significant improved by 40 points out of a 100-point scale. 10 out of 16 (63%) patients agreed that PRGF is better than any other treatments they have tried. Three out of 16 (19%) patients had complete resolution of symptoms with PRGF. This level of symptom improvement is clinically significant for such a refractory disease. Although not statistically significant, three out of five patients who had both treatments preferred PRGF over AST.

There was no change in objective measures, including visual acuity and cornea exam findings. This is not surprising because all patients had no evidence of tear dysfunction at baseline and near 20/20 vision. Confocal microscopy can image the corneal subepithelial nerve plexus, but it is not routinely performed at our center to diagnose keratoneuralgia because of lack of standardized protocols for performing the exam and analyzing the images. It is user dependent and potentially insensitive, because only a limited area of the subepithelial nerve plexus is imaged (22). Nerve abnormalities are also not specific for keratoneuralgia (23). Therefore, this technology is only performed on a subset of patients in our center who requested the procedure. It is interesting to note, however, that almost half of the patients replaced anti-inflammatory drops, including steroids, with suggesting it has anti-inflammatory activity. Since side effects have not been reported with PRGF, this suggests PRGF may be used in lieu of other anti-inflammatory treatments in ocular surface disease and corneal pain, especially if there are any medical contraindications or side effects with the latter.

The mechanism for plasma products in keratoneuralgia is likely multifactorial. Animal models have shown that platelet-rich plasma (similar to PRGF but without leukocyte filtration) can induce a potent antinociceptive effect by activating peripheral cannabinoid receptors to induce an analgesic effect (24). *In vitro* studies have also shown platelet-rich plasma can downregulate the NF-kB pathway which regulates expression of numerous inflammatory mediators, including chemotactic and neurosensitizing agents that could contribute to pain (25, 26). It also contains various concentrated growth factors that are postulated to regenerate abnormal corneal nerves (27). Tear immunoassays could be used to measure concentrations of inflammatory mediators and *In vivo* confocal microscopy has the potential to document changes in corneal inflammatory cells and nerve morphology in the future because it has been used to document nerve regeneration after AST use (9).

There are several limitations to this study. This is an observational study specifically capturing patient satisfaction with PRGF and did not utilize controls or comparisons with other treatments. The retrospectively administered questionnaire is subject to recall and sampling error, however, our comparison of the recalled initial pain scores from the questionnaire with the scores from chart review was not statistically significant. Our sample size, although small, included all patients since integrating PRGF into our treatment plan 5 years ago.

All blood products require aseptic technique, phlebotomy and technician personnel to manufacture the treatment, as well as careful transport and appropriate storage by patient. These barriers are compounded by cost, as blood-derived products are not reimbursed by insurance at this time. It is interesting to note that although 75% of our patients indicated that the cost of PRGF was too high, only 25% reported that the cost is preventing them from using PRGF. Other topical blood products, such as AST, have also reported efficacy for treating keratoneuralgia (8). Our center chose to use PRGF instead because PRGF has higher concentration of growth factors, a process to remove leukocytes 11, and is prepared using a FDA and EU approved commercial kit that can be prepared in the same standardized fashion at other centers.

Despite limitations and small sample size, our retrospective observational study is the first to demonstrate improvement in subjective symptoms in patients with keratoneuralgia treated with PRGF. Prospective and comparison studies to other treatments are needed to validate the therapeutic role of PRGF in treating this disease that is both difficult to diagnose and heal.

## Data availability statement

The original contributions presented in the study are included in the article/Supplementary material, further inquiries can be directed to the corresponding author.

## Ethics statement

The studies involving human participants were reviewed and approved by Baylor College of Medicine Institutional Review Board. The patients/participants provided their written informed consent to participate in this study.

## Author contributions

All authors contributed to this manuscript, including data review, data analysis, and drafting the manuscript.
