# Supplementary material for: Initial experiences using plasma rich in growth factors to treat keratoneuralgia
Source: Front Med (Lausanne). 2022 Aug 24;9:946828. doi: 10.3389/fmed.2022.946828 (PMC9448984; doi:10.3389/fmed.2022.946828)
Supplement: Supplementary file 1 [file Table_1.DOCX]

Supplementary Material

**Supplementary Table 1.** Telephone Questionnaire. Questions 2-7 are asked with qualifier, “for your eye pain”.

| **1** | **Are you still using Plasma Rich in Growth Factor drops?** |
| --- | --- |
|  | If you stopped, why did you stop? |
|  | If you stopped, how long did you use it? |
| **2** | **Rate the severity of your dry eye (0 no symptoms, 100 worst).** |
|  | Before you started plasma drops? |
|  | After you started using plasma drops? |
| **3** | **Rate the frequency of your dry eye (0 none, 100 all the time).** |
|  | Before you started plasma drops? |
|  | After you started using plasma drops? |
| **4** | **Are plasma drops worse than, equal to, or better than previous treatments?** |
| **5** | **Did you experience any side effects from plasma tears** |
|  | If so, what were those side effects? |
| **6** | **Would you recommend plasma drops to other patients?** |
|  | - If you would not recommend them, why? |
| **7** | **Are the cost of plasma drops acceptable or too high?** |
|  | Does the cost prevent you from continuing to use plasma? |
| **8** | **Do you use your plasma drops inside the bowl, as a normal eye drop, or both?** |
|  |  |
